# A pharmacokinetics–pharmacodynamics study of single-dose total glucosides of paeony capsule on reducing serum total bile acid in hepatic injury rats

**DOI:** 10.1080/13880209.2021.1937232

**Published:** 2021-06-21

**Authors:** Ninghua Jiang, Bohong Zheng, Yihan Feng, Lei Yin, Yuanrong Liu, Lujing Cao, Ning Zheng, Suxiang Wu, Baoyue Ding, Xuan Huang, Jeffrey Wang, Shuyu Zhan

**Affiliations:** a Department of Pharmacy, The Second Affiliated Hospital of Jiaxing University, Jiaxing, China; b Department of Pharmacy, College of Medicine, Jiaxing University, Jiaxing, China; c College of Pharmaceutical Science, Zhejiang Chinese Medical University, Hangzhou, China; d Department of Pharmaceutical Sciences, College of Pharmacy, Western University of Health Sciences, Pomona, CA, USA

**Keywords:** Paeoniflorin, albiflorin, hepatoprotection, serum biomarker, effect index, TBA, concentration–effect relationship

## Abstract

**Context:**

Total Glucosides of Paeony (TGP) capsule possesses various hepatoprotective activities. No study is available concerning TGP’s concentration–effect relationship on hepatoprotection.

**Objective:**

To establish a pharmacokinetics–pharmacodynamics (PK-PD) modelling on TGP capsule’s hepatoprotection after a single oral administration in hepatic injury rats.

**Materials and methods:**

Male Sprague-Dawley rats were divided into five groups (*n* = 6): control, model (hepatic injury), treated-H (2.82 g/kg), treated-M (1.41 g/kg), and treated-L (0.705 g/kg) groups. All treated groups rats were intragastrically administered a single dose. An LC-MS/MS method was applied to determine paeoniflorin (Pae) and albiflorin (Alb) in rat serum. The effects of single-dose TGP on serum alanine transaminase (ALT), aspartate transaminase (AST) and total bile acid (TBA) were evaluated in hepatic injury rats.

**Results:**

Single dose (2.82, 1.41, or 0.705 g/kg) TGP capsule could real-time down-regulate serum TBA but not ALT and AST in hepatic injury rats within 20 h. An inhibitory effect Sigmoid *E*_max_ of PK-PD modelling was established using Pae and Alb as PK markers and serum TBA as effect index. Pharmacodynamic parameters were calculated. For treated-H, treated-M and treated-L group, respectively, *E_0_* were 158.1, 226.9 and 245.4 μmol/L for Pae, 146.1, 92.9 and 138.4 μmol/L for Alb, *E_max_* were 53.0, 66.0, and 97.1 μmol/L for Pae, 117.4, 249.7 and 60.0 μmol/L for Alb, and *EC_50_* were 9.3, 5.2 and 2.7 μg/mL for Pae, 2.3, 0.8, and 0.8 μg/mL for Alb.

**Discussion and conclusions:**

Serum TBA is a sensitive effect index for TGP’s single dose PK-PD modelling, and it is potential for further multi-dose studies of TGP’ effect on hepatic injury. The study provides valuable information for TGP’s mechanistic research and rational clinical application.

## Introduction

**Figure 1.**
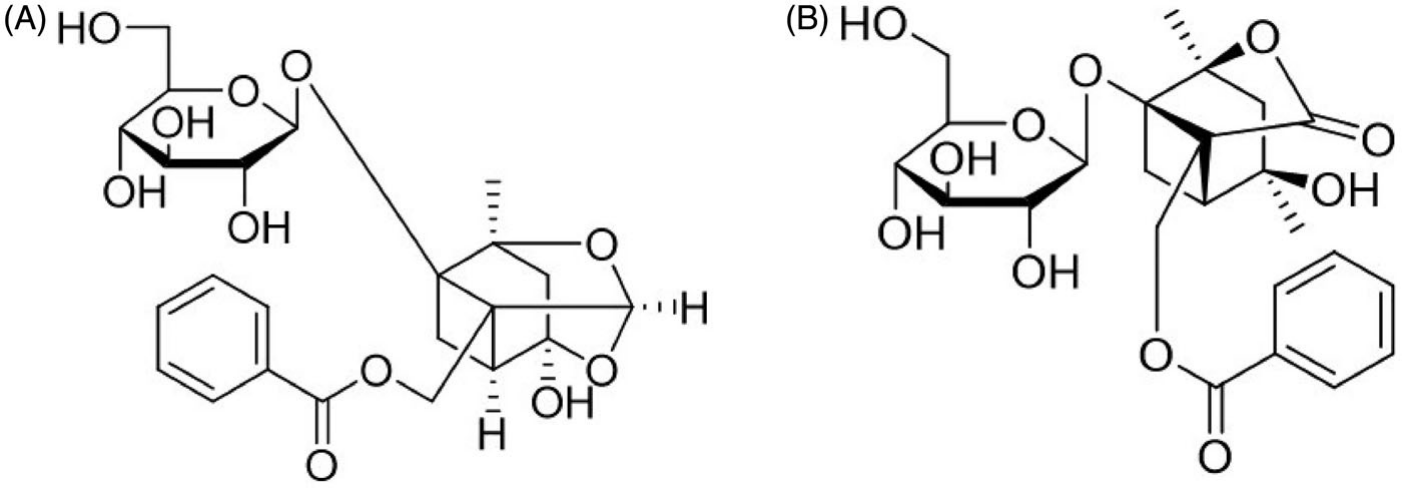
The structure of paeoniflorin (A, C_23_H_28_O_11_, 480.45) and albiflorin (B, C_23_H_28_O_11_, 480.45).

Radix Paeoniae Alba, a popular Traditional Chinese Medicine (TCM) derived from the dried root of *Paeonia lactiflora* Pall. (Paeoniaceae), has been used in China, Korea, and Japan for more than 1200 years (He and Dai 2011). Total Glucosides of Paeony (TGP), the main active substance extracted from Radix Paeoniae Alba, exhibits various pharmacological effects including anti-inflammatory, immunoregulatory, and antioxidative (He and Dai 2011; Zhao et al. 2013; Zheng et al. 2019), and it has been developed as a clinical preparation, TGP capsule, for the treatment of rheumatoid arthritis in China (Luo et al. 2017; Huang et al. 2019). TGP contains the ingredients of monoterpenoid glycosides in which paeoniflorin (Pae) and albiflorin (Alb) (Figure 1) are the main ingredients, and they account for the pharmacological effects observed for TGP in both *in vitro* and *in vivo* studies (Tabata et al. 2001; Chen et al. 2006; Wang et al. 2014). Therefore, Pae and Alb are usually designated as markers in TGP’s pharmacokinetic studies using normal or pathological animal models (Jiang et al. 2012; Gong et al. 2015; Fei et al. 2016). In recent years, the hepatoprotective activities of TGP including its protection against acute liver injury, non-alcoholic fatty liver diseases, chronic liver fibrosis and liver cancer have been well studied (Song et al. 2015; Li et al. 2017; Ma et al. 2017; Hu et al. 2018). Pharmacokinetics research has clarified TGP’s absorption, distribution, metabolism and excretion *in vivo* (Takeda et al. 1995; Fei et al. 2016; Wang et al. 2017; Zhu et al. 2018). However, the study of TGP’s concentration–effect relationship which would be significant for the drug’s development and rational clinical application is still lacking.

Pharmacokinetics–pharmacodynamics (PK-PD) modelling combines the profiles of the drugs’ concentration-time and effect-time to characterize their concentration–effect relationships *in vivo*. The successful PK-PD modelling with its PD parameters will reveal the drugs’ triggering effect and action time *in vivo* and helps to understand drugs’ pharmacological mechanisms and optimize dosage regimens. Therefore, PK-PD modelling has been widely used in drugs’ preclinical and clinical *in vivo* processes studies (Zhang et al. 2016). Nevertheless, it is critical to select and test suitable effect index which might be simply sampling and quantitative, and most importantly, related to drug’s pharmacological activities and real-time respond on the drug’s intervention (Agoram and van der Graaf 2012; Zhan et al. 2018). Serum biomarker is a more available effect index than others for pharmacodynamic study in PK-PD modelling because they can indirectly reflect drug’s activities as well as be synchronously monitored with drug concentrations in blood. Serum alanine transaminase (ALT), aspartate transaminase (AST) and total bile acid (TBA) are common and standard biomarkers applied in TGP’s hepatoprotection (Qin and Tian 2011; Ma et al. 2015, 2016). However, their real-time responses on single dose of TGP intervention remain unknown.

To select serum effect index for TGP’s PK-PD modelling establishment, this study investigates the real-time responses of serum ALT, AST and TBA on single oral TGP capsule intervention in carbon tetrachloride (CCl_4_)-induced hepatic injury rats through their effect–time relationships analysis. A simple and reliable LC-MS/MS method was applied to determine Pae and Alb in rat serum and subsequently used for pharmacokinetics study after single oral administration of TGP capsule in CCl_4_-induced hepatic injury rats. Meanwhile, serum effect indices of ALT, AST and TBA were evaluated at 3, 8, and 20 h after dosing, and pharmacodynamic profiles were constructed through effect-time relationship. As a result, serum TBA exhibited real-time response on single dose of TGP capsule intervention, and it was selected as a suitable effect index to establish an inhibitory effect PK-PD modelling of TGP’s hepatoprotection. This study helps set forth single-dose TGP’s concentration–effect relationship *in vivo* and would provide valuable information for TGP’s further mechanism studies on hepatoprotection and its clinical application.

## Materials and methods

### Chemicals and reagents

Pae (Lot: 180526), Alb (Lot: 171226) and gentiopicroside (internal standard, IS, Lot: 171106) standards were purchased from Shanghai Winherb Medical S & T Development Co. Ltd (Shanghai, China). TGP Capsule (Lot: 180515) containing 0.312 g/g paeoniflorin and 0.165 g/g albiflorin was provided by Lansen Ltd. (Ningbo, China). ALT, AST and TBA assay kits were purchased from Nanjing Jiancheng Bioengineering Institute (Nanjing, China). Water was produced by Milli-Q water system (Millipore, Bedford, MA, USA). Acetonitrile, methanol (Merck, Germany), and formic acid (Tedia, USA) were of HPLC grade. All other reagents were obtained from commercial sources and were of analytical grade.

### Instruments and LC-MS/MS conditions

The HPLC system consisted of a Waters e2695 liquid chromatography system, equipped with a binary pump, a vacuum degasser unit and an autosampler. Chromatographic separation was achieved on an Agilent Eclipse XDB C_18_ column (2.1 × 150 mm, 5 µm). The column temperature was set at 40 °C. An isocratic elution consisted of 0.1% formic acid-acetonitrile (85:15, v/v) with a flow rate of 0.3 mL/min was used for the separation. The sample injection volume was 20 μL.

Mass spectrometric signals were acquired using the Waters Acquity TQD tandem mass spectrometer (Waters, Manchester, UK) equipped with an electrospray ionisation (ESI) source. Electrospray capillary voltage was 3.6 kV, desolvation gas flow and temperature were maintained at 600 L/h and 380 °C, respectively, cone gas flow was maintained at 50 L/h, collision gas flow was 0.30 mL/min and the source temperature was 120 °C. The mass spectrometer was operated in the negative mode. Quantification was obtained using the multiple reaction monitoring (MRM) acquisition mode with a dwell time of 0.10 s by monitoring the precursor ion to product ion transitions of m/z 525.4→449.2 at 4.34 min for Pae, *m*/*z* 525.4→479.2 at 3.71 min for Alb, and *m*/*z* 401.1→179.1 at 3.05 min for the IS. The optimized collision energy values of −12 V and −10 V were chosen for the two analytes and IS, respectively. The cone voltage values of −26 V, −30 V, and −28 V were chosen for Pae, Alb and the IS, respectively. MassLynx version 4.1 software was used to operate the mass spectrometer, coupled with TargetLynx data processing software. Least squares linear regression and 1/x weighting were used to generate standard curves and peak area was used for peak quantification.

### Preparation of calibration standards and quality control samples

The methods of calibration and quality control samples preparations referred to our previous report (Zhan et al. 2015). In detail, the stock solutions of Pae (4.31 mg/mL) and Alb (2.22 mg/mL) were prepared in methanol. Then, the appropriate amounts of the two stock solutions were mixed and diluted with methanol to a final mixed standard solution. A series of working solutions of two analytes were obtained by diluting the mixed standard solution with methanol at appropriate concentrations. The IS stock solution (4.36 mg/mL) was also prepared in the methanol. All solutions were stored at 4 °C. To prepare calibration curves, each working solution (40 μL) was evaporated to dryness in an Eppendorf concentrator 5301 (Eppendorf, Germany). The residue was then spiked with a 40 μL drug-free serum sample and mixed to form calibration standards. To validate the method, high-, middle-, and low-quality control (QC) samples containing the two analytes were prepared using the stock solutions diluted to achieve Pae and Alb concentrations of 30.0, 6.00, 0.60 μg/mL and 15.0, 3.00, 0.30 μg/mL, respectively, and then were processed in the same manner as the calibration standard samples. The calibration standard and QC samples were prepared for each analysis batch.

### Experiments protocols

The animal experiments (JUMC2018-006) were performed strictly in accordance with the Principles of Animal Care and Use approved by The Animal Ethic Review Committee of Jiaxing University. Male Sprague-Dawley rats, weighting 200–250 g, were purchased from the Experiment Animal Centre of Zhejiang Province (Hangzhou, China; certificate number: SCXK 2017-0001). The rats were maintained in an air-conditioned animal quarter at a temperature of 22 ± 2 °C and a relative humidity of 50 ± 10% under a 12-h light/dark cycle for 7 days and then fasted with free access to water for 12 h prior to the experiment. Five groups of rats were included in the study (6 rats per group): High-dose drug-treated (treated-H) group, middle-dose drug (treated-M) and low-dose drug-treated (treated-L) group, model group, and control group. Except for the control group, each rat was intraperitoneally injected with 5 mL/kg CCl_4_ solution (10% in soybean oil). Twenty-four hours later, treated-H group, treated-M and treated-L group rats were intragastrically administered with TGP solutions (dissolving TGP capsule in saline aqueous) at the dose of 2.82, 1.41 and 0.705 g/kg, respectively. Rats in the model group were intragastrically administered equal volume of normal saline. Rats in the control group were intraperitoneally injected with normal saline and then 24 h later intragastrically administered with normal saline. For each group of rats, 200 μL blood samples were collected in Eppendorf tube via fossa orbitalis vein before modelling of CCl_4_ injection, before drug administration and subsequently at 5, 10, 20, 30, 60, 90, 120, 180, 240, 360, 480, and 720 min following administration. The samples were centrifuged at 5000 rpm, 4 °C for 10 min, and then, the serum samples were obtained, divided into aliquots and frozen at −80 °C until analysis.

### Sample preparation

For Pae and Alb analysis in serum, the serum samples were extracted using a liquid–liquid extraction technique. To each tube containing 40 μL serum, 1.0 mL ethyl acetate containing 1.00 μg/mL IS was added. The mixture was vortex-mixed for 5 min at 1500 rpm using a vortex mixer (IKA, Germany) and centrifuged for 5 min at 10,000 rpm. Next, 900 μL of supernatant was removed and placed into a new tube and evaporated at 40 °C to dryness in the Eppendorf concentrator. The residue was reconstituted in 80 μL acetonitrile/water (1:4, v/v) and centrifuged for 5 min at 10,000 rpm, and a 20.0 μL aliquot was injected for the LC-MS/MS analysis within 12 h. The serum biochemical index levels of ALT, AST and TBA were assayed by commercial test kits. Analysis was conducted using a Thermo Scientific Multiskan GO 1510 analyser (Thermo Fisher Scientific Oy Ratastie 2, Finland).

### Data analysis

Data were expressed as the mean ± standard deviation (SD). Pharmacokinetics parameters were estimated by non-compartmental analysis model and PK-PD modelling was established by pharmacodynamic compartmental model using WinNonlin 5.3 (Pharsight Corporation, Mountain View, CA). Goodness of fit was synthetically evaluated by the Akaike Information Criterion (AIC), Sum of Squares due to Regression (SSR) and correlatability of observed and predicted values (CORR). Differences between groups were evaluated by an unpaired Student’s *t*-test and *p* < 0.05 were considered statistically significant.

## Results and discussion

### Paeoniflorin and albiflorin determination in TGP capsule

**Figure 2.**
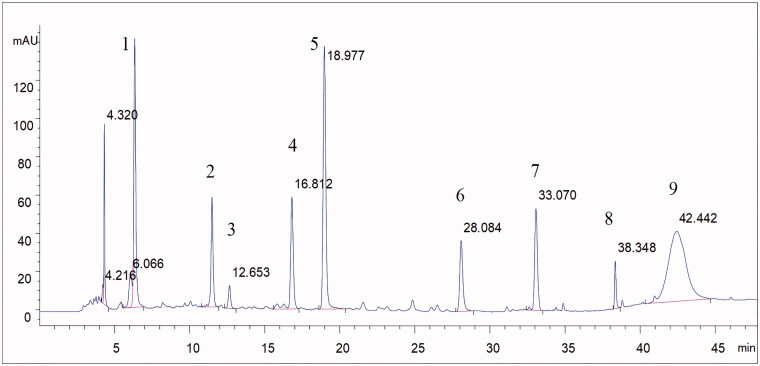
The full chromatographic spectrum of TGP (1. Gallic acid; 2. Hydroxypaeoniflorin; 3. Catechin; 4. Albiflorin; 5. Paeoniflorin; 6. Pentagalloyl glucose; 7. Benzoic acid; 8. Benzoylpaeoniflorin; 9. Paeonol).

To calculate administration dose of Pae and Alb, we determined these two analytes by HPLC method. Chromatographic separation was achieved using a C18 column (250 mm × 4.6 mm, 5 µm) operated at 30 °C with a flow rate of 0.8 mL/min. The mobile phase was consisted of 0.1% phosphoric acid aqueous solution (A) and acetonitrile (C) using a gradient elution of 10–15% C at 0–5 min, 15–22% C at 5–25 min, 22–70% C at 25–45 min, 70–80% C at 45–46 min and 80% C at 46–48 min. DAD detection was performed at 230 nm. The sample injection volume was 10 µL. The analytes solution was prepared by dissolving TGP capsule in 80% methanol. At least nine compounds including Pae and Alb were identified in TGP from the full chromatographic spectrum, which were showed in Figure 2, Calibration solutions of Pae and Alb was prepared by dissolving both standards in 80% methanol to make calibration curves. Pae and Alb from three independent packaging of capsule in one batch were determined using their calibration curves by HPLC method. The determination results of Pae and Alb content in TGP capsule were 0.312 ± 0.008 and 0.165 ± 0.002 g/g, respectively.

### LC-MS/MS method validation

**Figure 3.**
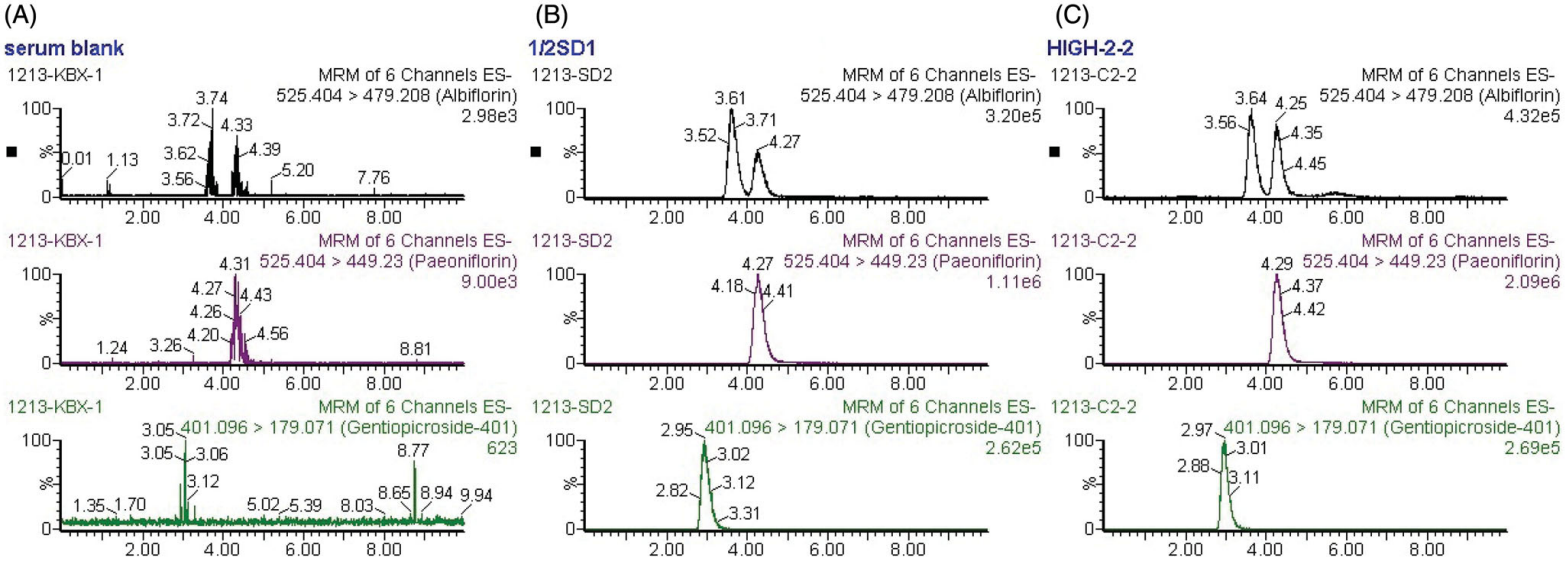
MRM chromatograms of paeoniflorin, albiflorin and gentiopicroside (IS). (A) blank serum; (B) blank serum spiked with paeoniflorin, albiflorin and IS (30, 15 and 1 μg/mL respectively; (C) rat serum collected at 10 min after administration of 2.82 g/kg TGP.

The specificity of LC-MS/MS method was evaluated, and no significant interferences from endogenous substances in the blank serum were observed at the retention times of Pae, Alb and IS (Figure 3). The method exhibited a highly linear response over the selected concentration range of 0.010–60.0 μg/mL for Pae and 0.005–30.0 μg/mL for Alb by weighted (1/*x*) least-squares linear regression analysis. The mean matrix effects, investigated by comparing the analytical response of the analytes spiked with the blank serum samples after extraction with those of pure standard solutions containing analytes at the same concentrations, were in the range of 97.1–99.9% for Pae and 77.6–99.1% for Alb, which indicated there is little matrix effect on the determination of Pae and Alb in rat serum. The intra- and inter-day precision and accuracy of the method were determined by replicating QC samples at three concentration levels. The mean accuracy of all concentrations was in the range of 87.8–112%, and the intra- and inter-day precisions ≤14.5% and ≤14.9%, respectively, for the two analytes (Table 1). The stability of the analytes in serum was assessed by analysing six replicates of QC samples at three concentration levels under different conditions, and it was shown in Table 2 that Pae and Alb were stable for 12 h in autosampler after sample preparation, in samples after three freeze–thaw cycles and in storage at −20 °C for 30 days with accuracy in the range of 88.7–113%. Therefore, this method is qualified for a routine pharmacokinetic study.

**Table 1.** Precision and accuracy for the assay of paeoniflorin and albiflorin in rat serum (*n* = 6).

Analyte and concentration spiked (μg/mL)	Intra-day			Inter-day		
	Concentration measured (μg/mL)	Accuracy (%)	Precision (%)	Concentration measured (μg/mL)	Accuracy (%)	Precision (%)
Paeoniflorin						
0.60	0.67 ± 0.09	112	13.8	0.64 ± 0.06	106	10.0
6.00	6.54 ± 0.94	109	14.3	5.90 ± 0.66	98.3	11.1
30.0	26.3 ± 1.59	87.8	6.0	30.8 ± 3.50	103	11.4
Albiflorin						
0.30	0.34 ± 0.04	112	12.9	0.30 ± 0.02	100	7.3
3.00	3.22 ± 0.47	107	14.5	2.96 ± 0.29	98.8	9.6
15.0	13.2 ± 0.68	87.8	5.2	14.7 ± 2.19	98.2	14.9

**Table 2.** Stability of paeoniflorin and albiflorin in rat serum and standard solutions (*n* = 6).

Analyte and concentration spiked (μg/mL)	In autosampler after preparation for 12 h		After three freeze-thaw cycles		At −20 °C for 30 days	
	Concentration measured (μg/mL)	Accuracy (%)	Concentration measured (μg/mL)	Accuracy (%)	Concentration measured (μg/mL)	Accuracy (%)
Paeoniflorin						
0.60	0.63 ± 0.05	105	0.56 ± 0.02	92.7	0.62 ± 0.02	103
6.00	6.01 ± 0.72	100	6.20 ± 0.51	103	5.81 ± 0.20	96.9
30.0	31.7 ± 2.17	106	34.0 ± 1.79	113	30.4 ± 0.58	101
Albiflorin						
0.30	0.27 ± 0.01	88.7	0.29 ± 0.03	97.1	0.28 ± 0.03	95.0
3.00	3.05 ± 0.40	102	3.18 ± 0.08	106	2.88 ± 0.30	96.0
15.0	16.9 ± 0.90	113	16.3 ± 0.53	109	14.2 ± 0.71	94.6

### Pharmacokinetic profiles

**Figure 4.**
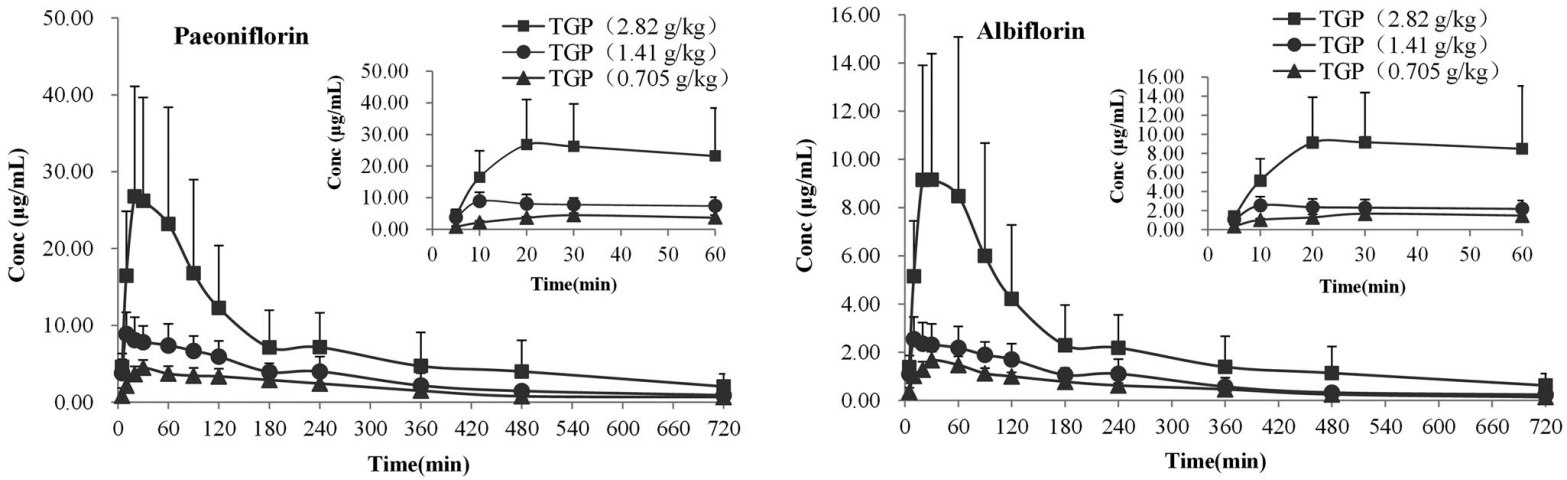
Mean serum concentration–time curves of paeoniflorin (A) and albiflorin (B) after intragastric administration of 2.82, 1.41 and 0.705 g/kg TGP in CCl_4_-induced hepatic injury rats (*n* = 6).

The serum concentrations of Pae and Alb in hepatic injury rats after single intragastric administration of 2.82, 1.41 and 0.705 g/kg TGP were determined, respectively, by the validated LC-MS/MS method. The mean serum concentration-time curves of the two analytes are illustrated in Figure 4. The pharmacokinetic parameters based on non-compartmental analysis are summarized in Table 3. The pharmacokinetic results showed that for PK markers of Pae and Alb, their main parameters of *T*_max_, *t*_1/2_ and MRT_0–∞_ had definitely no significant differences (*p* > 0.05) at three oral doses, which demonstrated they display similar pharmacokinetic characteristics *in vivo*. It was interesting finding that the mean *t*_1/2_ and MRT_0–∞_ of the two analytes were about 3 h and 5 h which are definitely higher than other pharmacokinetics study of Pae and Alb in normal rats (about 2 h and 3 h) (Fei et al. 2016). These results suggested pathological conditions of hepatic injury might prolong residence time of Pae and Alb *in vivo* which also been reported in other researches (Jiang et al. 2012; Xiong and Wang 2016; Xu et al. 2016). It is well known that the liver is an important organ for drug biotransformation and metabolism especially because it contains the enzyme of cytochrome P450 which plays vital role in converting original drugs into their metabolites (Chiu et al. 2018; Wang et al. 2018). Hepatic injury changes cytochrome P450 isoenzyme contents, which could alter the disposition of drugs in the body (Xie et al. 2014; Li et al. 2017). Besides, hepatic injury could influence intestinal drug transport and caused the disorder of the intestinal microbiota (Li et al. 2010; Fouts et al. 2012). Taken together, all these might cause the differences in pharmacokinetic behaviour of Pae and Alb between hepatic injury and normal rats after oral administration of TGP. Therefore, it should draw our attention to TGP’s clinical rational application on hepatoprotection although the underlying effect of hepatic injury on TGP’s pharmacokinetic behaviour still needs further research.

**Table 3.** Pharmacokinetic parameters of paeoniflorin and albiflorin in CCl_4_-induced hepatic injury rats after intragastric administration of 2.82, 1.41 and 0.705 g/kg TGP (*n* = 6).

Parameter (units)	2.82 g/kg		1.41 g/kg		0.705 g/kg	
	Paeoniflorin	Albiflorin	Paeoniflorin	Albiflorin	Paeoniflorin	Albiflorin
C_max_ (μg/mL)	28.6 ± 15.1	10.4 ± 6.0	9.3 ± 2.5	2.8 ± 0.8	4.4 ± 0.5	1.8 ± 0.3
T_max_ (min)	30.0 ± 15.5	30.0 ± 15.5	31.7 ± 29.9	26.7 ± 18.6	26.7 ± 5.8	40.0 ± 17.3
t_1/2_ (min)	206.4 ± 101.4	195.6 ± 84.2	192.8 ± 110.8	184.7 ± 85.4	260.6 ± 51.4	203.7 ± 51.1
MRT_0-∞_ (min)	316.2 ± 183.9	289.7 ± 142.4	297.3 ± 149.9	280.0 ± 134.2	362.8 ± 38.2	279.8 ± 53.6
AUC_0-t_ (min*μg/mL)	5299.2 ± 3513.2	1724.3 ± 1202.9	2170.0 ± 657.9	606.1 ± 206.1	1247.1 ± 227.1	401.1 ± 43.9
AUC_0-∞_ (min*μg/mL)	6081.6 ± 3890.1	1946.1 ± 1360.2	2449.4 ± 782.9	673.2 ± 229.2	1476.1 ± 312.7	439.6 ± 57.4
V_d_/F(mL/kg)	51.6 ± 25.6	82.1 ± 40.3	49.4 ± 26.2	93.8 ± 40.0	57.0 ± 12.6	82.7 ± 29.6
CL/F (mL/min/kg)	0.19 ± 0.10	0.32 ± 0.15	0.20 ± 0.06	0.38 ± 0.13	0.15 ± 0.03	0.28 ± 0.04

### Serum ALT, AST and TBA evaluation

**Figure 5.**
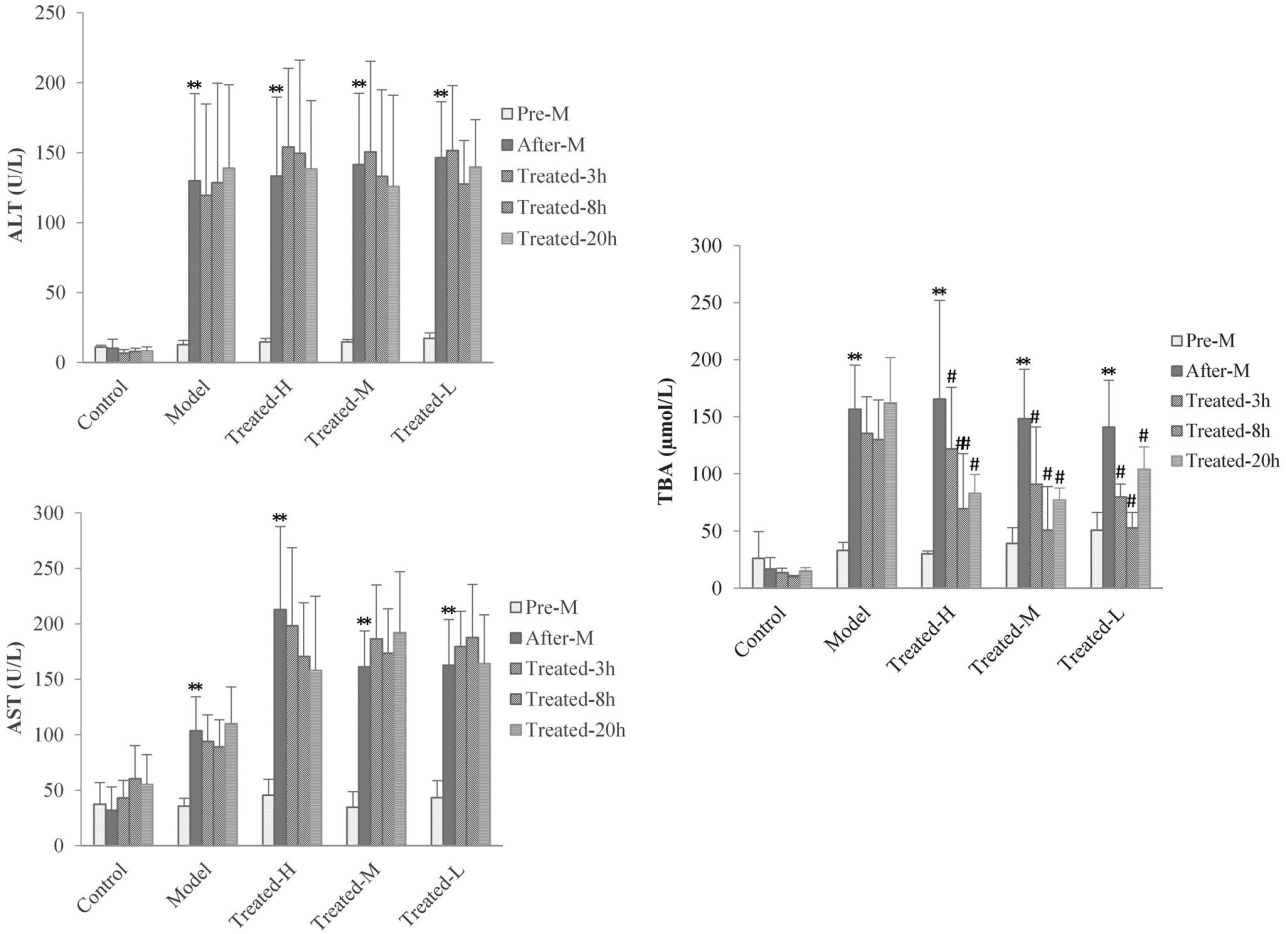
Serum ALT (A), AST (B) and TBA (C) levels in rats at pre-disease modelling (Pre-M), after-disease modelling (After-M), 3 h after drug administration (Treated-3 h), 8 h after drug administration (Treated-8 h) and 20 h after drug administration (Treated-20 h). (*n* = 6, **p < 0.01 vs. pre-M, ^#^*p* < 0.05, ^##^*p* < 0.01 vs. after-M).

To evaluate the real-time response of serum ALT, AST and TBA on single dose of TGP intervention in hepatic injury rats, we investigated the levels of ALT, AST and TBA in serum for all group rats at pre-disease modelling (pre-M), after-disease modelling (after-M) and at 3 h (treated-3 h), 8 h (treated-8 h) and at extra 20 h (treated-20 h) when the drugs have been completely eliminated after TGP administration, respectively. The results are shown in Figure 5. Compared to pre-disease modelling, the levels of serum ALT, AST and TBA after-disease modelling significantly increased in the model, treated-H and treated-L groups except for the control group, which meant these biomarker’s associated hepatic injury reflect induced by CCl_4_. Notably, compared with the control and model groups, in all treated groups, it was found that the levels of serum TBA were significantly down-regulated (*p* < 0.01 or *p* < 0.05) at 3, 8 and 20 h after drug administration, while the levels of serum ALT and AST had no significant changes (*p* > 0.05) at all these time points. These results implied single dose TGP intervention could affect serum TBA levels instantly but cannot down-regulate serum ALT and AST levels. It was well proved that TGP could simultaneously reverse abnormal elevation of serum ALT, AST and TBA in hepatic injury but the effect was based on repeat drug intervention for a few days (Qin and Tian 2011; Ma et al. 2015, 2016). Thus, the effect of TGP’s real-time regulation on these indices remains unknown and it should be firstly investigated under single dose administration. In this single dose effect-time investigation of TGP, it was interesting finding that serum TBA but not ALT and AST could sensitively respond to TGP’s intervention, which implied an underlying important TBA-related regulatory pathway in the mechanism of TGP’s hepatoprotection. TGP as well as its main components of Pae and Alb might alleviate the intrahepatic accumulation of toxic bile acids in CCl_4_-induced hepatic injury which has also been found in other liver disease such as cholestasis (Ma et al. 2016). Therefore, as a specific and sensitive diagnostic indicator for liver parenchymal injury and digestive system diseases (Shima et al. 2000; Hotta et al. 2005; Dawson 2017), TBA might also serve as a potential sensitive effect index for the PD profile and PK-PD modelling studies of TGP’s hepatoprotection.

### Pharmacodynamics profiles

**Figure 6.**
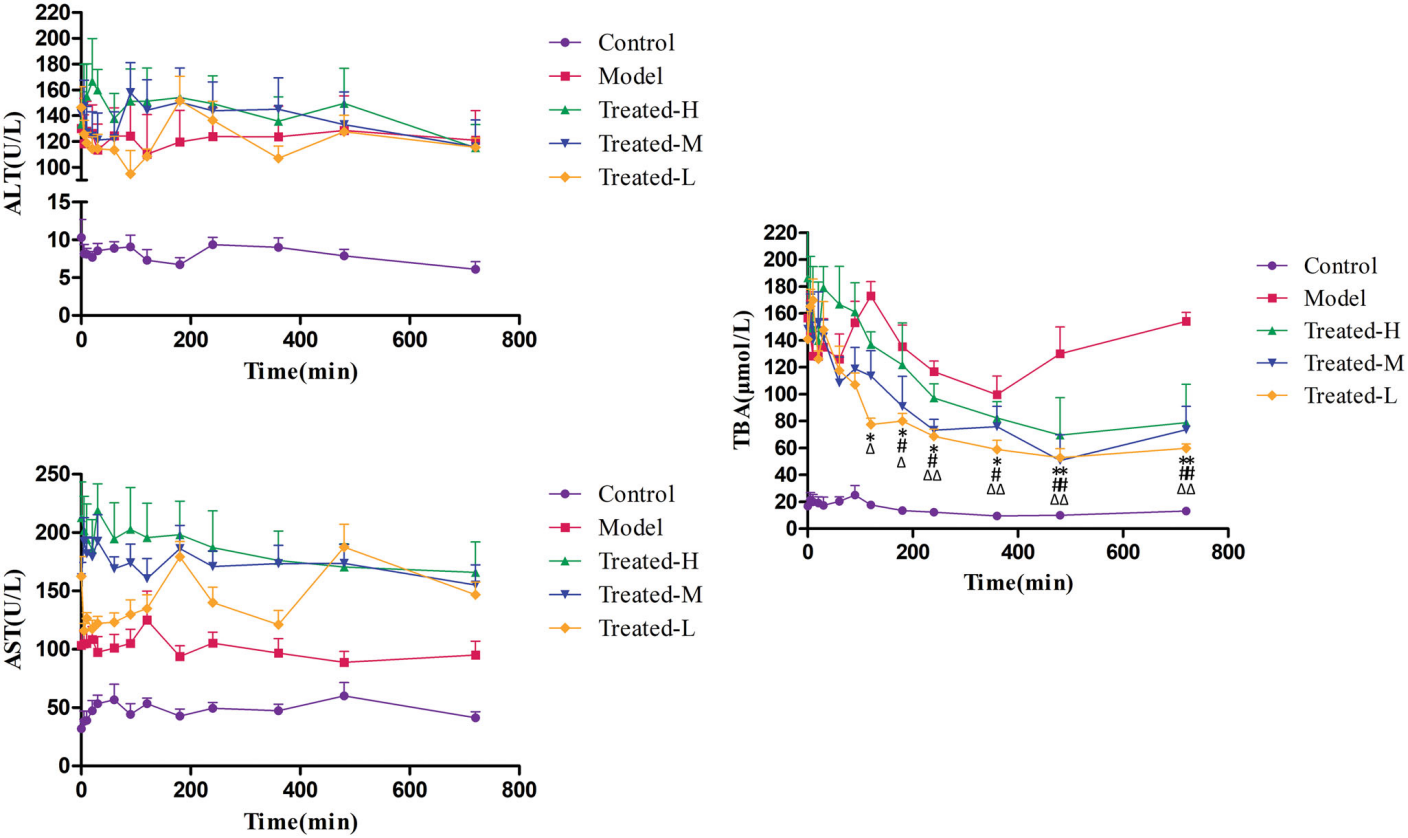
Serum ALT (A), AST (B) and TBA (C) levels over time in the Control, Model, Treated-H, Treated-M and Treated-L groups after single dose administration, respectively (*n* = 6). Treated-H group: *p < 0.05, **p < 0.01 vs. Time = 0; Treated-M group: ^#^*p* < 0.05, ^##^*p* < 0.01 vs. Time = 0; Treated-L group: ^Δ^*p* < 0.05, ^ΔΔ^*p* < 0.01 vs. Time = 0.

For PK-PD modelling, it is necessary to find regular response of effect index after drug intervention, which benefits the setup of pharmacodynamic profiles and the subsequent related analysis with drug concentration. Consequently, based on the results of real-time serum ALT, AST and TBA evaluation, we further determined their levels in serum at each time point within 12 h and then analysed the effect–time relationships. The serum ALT, AST and TBA levels with the time after administration for control group, model group, treated-H group, treated-M group and treated-L group were shown in Figure 6. It was found that serum ALT and AST levels stayed relatively stable over the time after single administration of TGP in all treated groups, which indicated single-dose TGP could not cause instant responses of serum ALT and AST within 12 h. In contrast, serum TBA showed significant decrease at each time point from 120 min or 180 min to 720 min after TGP administrations when compared with before administrations (Time = 0) for treated-H group, treated-M group and treated-L group, respectively (*p* < 0.01 or *p* < 0.05). Obviously, it was a regular response that serum TBA decreased gradually with time after TGP administration, so TBA could be related to Pae and Alb concentrations in serum for concentration–effect relationship analysis of TGP in hepatic injury rats.

### Pharmacokinetics–pharmacodynamics modelling

Based on the inhibitory effect of TGP on serum TBA from the above results, an inhibitory effect model was chosen to describe the effect–concentration relationship using Pae and Alb as PK markers and serum TBA as effect index. The PK-PD modelling was established using pharmacodynamic compartmental model in WinNonlin 5.3. When compared to the inhibitory effect *E*_max_ model, the inhibitory effect Sigmoid *E*_max_ model was found to have better fitness for the PK-PD data using the following equation (Equation (1)):

(1) $$E=E_{\max}-\frac{(E_{\max}-E_{0})C^{\gamma }}{C^{\gamma }+{\mathrm{EC}}_{50}^{\gamma }}$$

where *E* is the effect of serum TBA level, *E_0_* the initial effect of serum TBA level, *E_max_* the maximal effect, *EC_50_* the drugs concentration eliciting a half-maximal effect and *γ* the midpoint slop of the curve, a shape coefficient that describes the sensitivity of the concentration–effect relationship.

**Figure 7.**
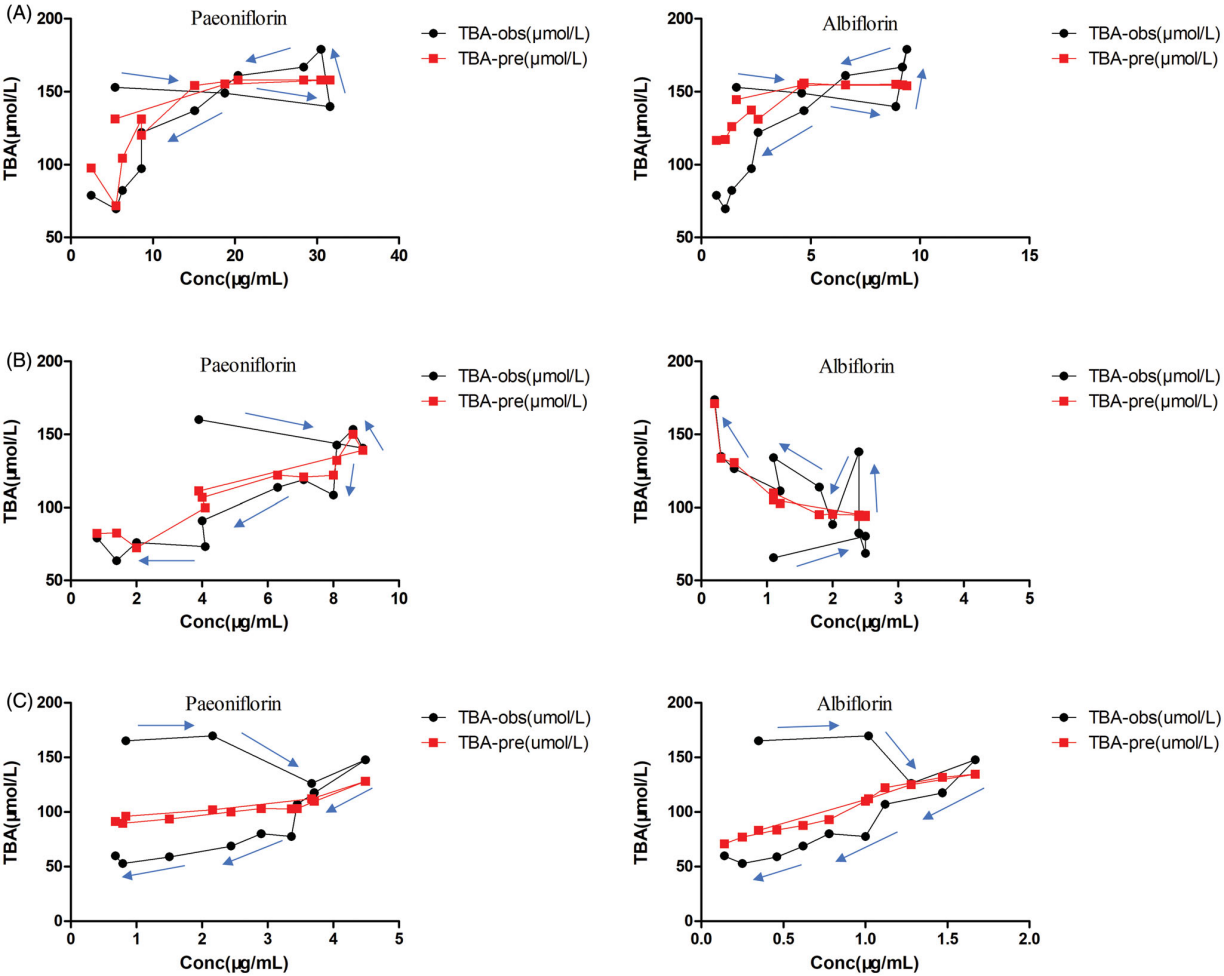
The observed mean serum TBA levels effect (TBA-obs) and the predicted mean serum TBA levels effect (TBA-pre) vs paeoniflorin and albiflorin concentration after oral administration of 2.82 (A), 1.41 (B) and 0.705 g/kg (C) TGP in CCl_4_-induced acute hepatic injury rats.

The mean PD parameters of Pae and Alb calculated using Equation (1) were summarised in Table 4. According to these PK-PD models, the predicted mean effects of TGP on reducing serum TBA levels in hepatic injury rats were consistent with the observed mean effects (Figure 7), which indicated these models’ acceptable prediction ability. The established models could be used to predict TGP’s effect on real-time down-regulation of serum TBA in hepatic injury rats based on their concentration-effect relationships.

**Table 4.** Mean pharmacodynamic parameters of paeoniflorin and albiflorin for reducing serum TBA levels effect after oral administration of 2.82, 1.41 and 0.705 g/kg TGP in CCl_4_-induced acute hepatic injury rats.

Parameters	2.82 g/kg		1.41 g/kg		0.705 g/kg	
	paeoniflorin	albiflorin	paeoniflorin	albiflorin	paeoniflorin	albiflorin
*E*_max_ (μmol/L)	53.0 ± 59.4	117.4 ± 123.9	66.0 ± 44.5	249.7 ± 153.6	97.1 ± 59.0	60.0 ± 38.1
*EC*_50_ (μg/mL)	9.3 ± 8.8	2.3 ± 2.9	5.2 ± 3.5	0.8 ± 0.9	2.7 ± 2.3	0.8 ± 0.3
*E*_0_ (μmol/L)	158.1 ± 60.8	146.1 ± 42.9	226.9 ± 195.4	92.9 ± 19.1	245.4 ± 255.5	138.4 ± 19.3
*γ*	8.2 ± 3.6	5.0 ± 4.3	7.7 ± 2.6	6.4 ± 4.8	2.1 ± 3.6	6.0 ± 3.5

It is important to investigate suitable effect index which must be related to pharmacodynamics and drug concentration changes *in vivo* for successful PK-PD modelling establishment. Recently, the mechanism-based PK-PD modelling studies of traditional Chinese medicine (TCM) using biomarkers as substitute effect indices have been reported (Wang et al. 2017; Cheng et al. 2018; Zhan et al. 2018). However, studies on PK-PD modelling of TCMs are still very limited mostly because of the shortage of suitable effect index. TCMs possess characteristics of multi-component, multi-target and integrated function and it is often difficult to find definitely direct effect index after TCMs intervention in clinical treatment. Serum usually contains important biomarker which might be related to disease and TCM’s integrated regulation so it is valuable to search prospective effect index from serum biomarker to evaluate TCM’s function. Nevertheless, most of TCMs in clinic application take effect slowly and usually require multi-dose intervention to show significant effects, which implies the hysteresis response of effect indices. Therefore, it is necessary to perform detailed investigation of serum effect index for TCMs’ pharmacodynamics and PK-PD studies. In this study, we commenced the investigation of common serum effect indices in TGP’s hepatoprotection and found serum TBA as feasible index for TGP’s PK-PD modelling establishment when single-dose administration. It would be of value to define the associated mechanism of TGP’s hepatoprotection and provide valuable information for product development and rational clinical application of TGP. However, further research is needed and will be our following study to explore the concentration-effect relationships within more boarder dose ranges, and (or) compare the response of these serum indices on multi-dose TGP intervention.

## Conclusions

This study established a PK-PD modelling of TGP capsule’s effect on reducing serum TBA level in CCl_4_-induced acute hepatic injury rats. A specific and reliable LC-MS/MS method was developed to simultaneously determine serum paeoniflorin and albiflorin, and then applied to the pharmacokinetic study of TGP in hepatic injury rats. We evaluated the real-time effect of TGP on serum ALT, AST and TBA after single dose administration. It was found that single oral TGP administration in hepatic injury rats could real-time down-regulate serum TBA but not ALT and AST levels. Moreover, TGP’s inhibitory effect on serum TBA displayed certain concentration-effect relationship. Consequently, using serum Pae and Alb as PK markers, and TBA as effect index, we successfully established an inhibitory effect Sigmoid E_max_ of PK-PD modelling of TGP on reducing serum TBA level in hepatic injury rats. This study might be useful to elaborate the concentration-effect relationship for TGP’s hepatoprotection and provides valuable information for the further studies of TGP’s mechanism and rational clinical application.
